# Nonparametric Statistical Inference with an Emphasis on Information-Theoretic Methods

**DOI:** 10.3390/e24040553

**Published:** 2022-04-15

**Authors:** Jan Mielniczuk

**Affiliations:** 1 Institute of Computer Science, Polish Academy of Sciences, Jana Kazimierza 5, 01-248 Warsaw, Poland; miel@ipipan.waw.pl; 2 Faculty of Mathematics and Information Science, Warsaw University of Technology, Koszykowa 75, 00-662 Warsaw, Poland

The presented volume addresses some vital problems in contemporary statistical reasoning. One of them is high dimensionality of the studied phenomenon and its consequences for formal statistical inference. A huge number of studies have been devoted to proposing new solutions and/or to modifying existing ones in order to account for the specificity of high-dimensional data. However, frequently, these methods work well for precisely defined parametric models and fail when misspecification occurs. Thus, there is a growing need to develop non-parametric and robust procedures accounting for this problem and to study existing methods when misspecification is suspected. This has been discussed in several papers in this volume under various scenarios. Furthermore, information theoretic methods due to their generality are of special interest in this context, e.g., when variable selection is envisaged. Frequently, the approach to account for high-dimensionality is based on the penalization of classic statistical procedures, and this line of reasoning is discussed here. Moreover, in a multivariate scenario, there is a need to define and study analogues of statistical measures designed for the univariate or bivariate case, and this approach is represented by the study on tail dependence indices. The important area of statistical research is devoted to time series analysis, especially in multivariate cases and in non-standard observability scenarios; two papers in the volume address this issue. Furthermore, information theoretic tools used to shed a new light on the generalization risk in learnability theory are covered here.

In [1], the general class of non-stationary multivariate processes is considered based on *p*-dimensional Bernoulli shifts, which, in particular, encompass multivariate linear processes with time-varying coefficients. A locally stationary model is proposed, under which its covariance matrix Σ(t) is piecewise Lipschitz continuous except at a certain number of breaks (change points). The problem of the non-parametric estimation of change points is addressed as well as that of graph support recovery, specifically the estimation of the set $\left\{\left(j,k\right):|\Sigma {\left(t\right)}^{-1}\left(j,k\right)|>u\right\}$ for a given threshold *u* and precision matrix $\Sigma {\left(t\right)}^{-1}$. It is shown that in both problems, one can obtain theoretical guarantees of the accuracy of estimation procedures using the proposed kernel smoothed constrained $\ell_{1}$ minimization approach.

In [2], the problem of support recovery is considered for a semiparametric binary model in which the posterior probability of the response is given by $q(\beta^{T}x)$, where *q* is an unknown response function. The problem is dealt with by applying the penalized empirical risk minimization approach for a convex loss ϕ. This has nice information theoretic connotations when ϕ is a logistic loss, as, in this case, we aim at estimating the averaged Kullback–Leibler projection of $q(\beta^{T}x)$ on the family of logistic models. For a high-dimensional setting and random subgaussian regressors, the conditions are studied, under which the minimizer of penalized empirical risk $\hat{\beta }$ converges to vector $\beta^{*}$ corresponding to the Kullback–Leibler projection. This is used to establish selection consistency of the Generalized Information Criterion GIC based on $\hat{\beta }$ for Lipschitz and convex ϕ under Linear Regressions Conditions. The resulting Screeing and Selection (SS) procedure is studied in numerical experiments.

Ref. [3] addresses one of the main issues of the learnability theory, namely the properties of generalization risk for the given learning algorithm L. I. Alabdulmohsin introduces a new concept of the uniform generalization of L with a rate ε that stipulates that the generalization risk is less than ε for any bounded loss function l(·,·) such that l(·,h) depends on the underlying sample only through the hypothesis *h* chosen by L. The information-theoretic characterization of this property is given in terms of variational information $J(\hat{z},h)$ between a single observation $\hat{z}$ and chosen hypothesis *h* (Theorem 2). In Theorem 4, the probabilistic inequality for deviation of empirical risk from the true risk is given in terms of $J(\hat{z},h)$. Moreover, the concept of the learning capacity of L, analogous to the concept of Shannon channel capacity, is introduced and studied.

Ref. [4], similarly to [2], deals with the classification problem of a binary variable under misspecification. It focuses on establishing a general upper bound of excess risk, i.e., the difference between the risk of the linear classifier ${\hat{\beta }}^{T}x$, obtained as a minimizer of the penalized empirical risk pertaining to convex function ϕ, and the Bayes risk in such a case (Theorem 1). The crucial part of the bound is the probability that $|\hat{\beta }-\beta^{*}{|}_{1}$ exceeds a certain threshold, where $\beta^{*}$ is the minimizer of the theoretical risk pertaining to ϕ. Interestingly, the authors are able to bound this probability, provided the predictors are multivariate subgaussian, for non-Lipschitz quadratic risk $\phi \left(t\right)={\left(1-t\right)}^{2}$, which is rarely studied in the classification context. The second part of the paper deals with consistency of the thresholded Lasso selector under the Linear Regression Conditions mentioned above and again for quadratic loss. The result complements the results on selection consistency studied in [2].

The paper [5] is an insightful study of introduced tail dependence indices in the multivariate case from a novel perspective, which sheds a new light on their similarities and differences. Namely, a set of five natural properties are introduced, which should be satisfied by such indices, and existing proposals (Frahm’s extremal dependence, Li’s tail dependence and Schmid’s and Schmidt’s tail dependence measures) are investigated in this context. Further properties of these indices are studied such as their behavior with increasing dimensions of the vector. The delicate problem of estimating the tail indices is addressed, and the consistency of the introduced estimators is studied. Their performance is illustrated using the EURO STOXX 50 index.

Ref. [6] considers non-parametric variable selection based on information-theoretic criteria. In such an approach, the maximization of conditional mutual information $CMI=I(X,Y|X_{S})$ is often considered in greedy selection, where *Y* is the response, $X_{S}$ is a vector of already chosen predictors, and *X* is a candidate for a possible augmentation of $X_{S}$. Frequently, conditional mutual information is replaced by the approximations resulting from Möbius expansion or some modifications of these approximations. In the paper, two criteria obtained in such a way, namely Conditional Infomax Feature Extraction (CIFE) and Joint Mutual Information (JMI), are analyzed, together with CMI, in a certain dependence model called the Generative Tree Model. It is shown that the two considered criteria may lead to a different order of chosen variables than the order induced by CMI, and CIFE may disregard a significant part of active variables. The analysis is based on formulae for the entropy of the multivariate Gaussian mixture and its mutual information with mixing variables derived in the paper, which are interesting in their own right.

In [7], the authors consider a semiparametric stationary time series model of the form $Z_{t}=x_{t}^{T}\beta +f\left(s_{t}\right)+\varepsilon_{t}$, where $x_{t}$ is a vector of random explanatory variables, $s_{t}$ is a temporal covariate, and $\varepsilon_{t}$ is an autoregressive process. Moreover, $Z_{t}$ is subject to random censoring from the right, and *f* is a linear combination of B-spline basis functions of order *q* with a corresponding vector of coefficients α. The penalized adaptive spline approach is developed in the paper to tackle the data irregularity and is then applied to an unbiased synthetic transformation of $Z_{t}$. The bias and covariance structure of the obtained estimators of α and β are derived, and their consistency is studied.

Ref. [8] addresses practically important and intensively researched problem of accounting for outliers in the estimation process when fitting the multiple linear regression model. The approach is based on the L2E parametric method proposed by the first author, which consists of finding the minimizer of the estimated Integrated Squared Error (ISE) in a parametric family of densities {f(x|θ)}. The proposed extension introduces an additional parameter *w*, which loosely corresponds to the mixture proportion of the main (outlier-free) component of the density, and the minimization is now performed in family {wf(θ|x)} with respect to both θ and *x*. The authors then convincingly show by analyzing several examples that the proposed method yields a much more adequate fit of residuals than the least squares, and additional insight into data interpretation is sometimes possible.
